# Comorbidity in Multiple Sclerosis

**DOI:** 10.3389/fneur.2020.00851

**Published:** 2020-08-21

**Authors:** Melinda Magyari, Per Soelberg Sorensen

**Affiliations:** ^1^Department of Neurology, Danish Multiple Sclerosis Center, Copenhagen University Hospital, Rigshospitalet, Denmark; ^2^Department of Neurology, The Danish Multiple Sclerosis Registry, Copenhagen University Hospital, Rigshospitalet, Denmark

**Keywords:** registry, comorbidity [MeSH], multiple sclerosis, comorbidities, coexisting diseases

## Abstract

Comorbidities in patients with multiple sclerosis (MS) has become an area of increasing interest in the recent years. A comorbidity is defined as any additional disease that coexists in an individual with a given index disease and that is not an obvious complication of the index disease. The aim of this review is to describe the current evidence regarding the range of comorbidities in the population with MS reported in different countries and the current knowledge about the influence of comorbidities on the clinical features and therapeutic challenges in MS. Certain comorbidities are more prevalent in people with MS such as depression, anxiety, cerebro- and cardiovascular diseases, and certain autoimmune disorders such as diabetes, thyroid disease, and inflammatory bowel disease. A previous perception of a trend toward a lower overall risk of cancer in patients with MS appears to be challenged, but there is no evidence on any higher occurrence of malignancies in the population with MS. Comorbidities may modify the clinical presentation of MS, and have implications for treatment choice, adherence, and outcome. Several comorbid conditions are associated with increased disability progression, including diabetes, hypertension, and chronic obstructive pulmonary disease. Comorbidities are common in MS from the time of diagnosis and may account for some of the heterogeneity observed in MS, including diagnostic delay, clinical presentation, degree of disability progression, rate of health care utilization, working ability, employment status, and quality of life. Coexisting diseases and polypharmacy increase the complexity of patient management and poses major challenges, particularly with the increasing number of immunosuppressive disease-modifying therapies.

## Introduction

Comorbidity has become an area of increasing interest in recent years because it is highly relevant to clinical care in multiple sclerosis (MS).

Comorbidity can be defined as any additional disease that coexists in an individual with a given index disease, which is not an obvious complication of the index disease (1). Classic definitions of comorbidity do not include health behaviors. However, behaviors such as smoking, alcohol intake, and sedentary lifestyle affect the risks and also the outcomes of chronic diseases, including MS (2).

In a recent comprehensive review, Marrie et al. (3) presented the incidence and the prevalence of a variety of comorbidities in MS. The increasing awareness on comorbidities influencing different aspects of MS has resulted in several approaches to address the gap of knowledge related to this topic. There is a considerable variability across studies from different geographical regions as to the study population and the methods used to study comorbidities. However, during the last 5 years, the scientific methods used to study comorbidities and their consequences have been improved as recent studies have been population-based or have included large populations trying to provide valid estimates.

Overall, however, it seems that comorbidities influence the course of MS and has medical and socioeconomic consequences for persons with MS (4).

It is generally accepted that the risk of multi-morbidity increases with age and that the risk is increased in patients with a chronic disease such as MS. During the last decades, the MS population has been aging in parallel with increasing general life expectancy and also as a result of improved disease modifying treatments (DMTs) (5). Polypharmacy due to medical treatment of comorbidities may also obscure the clinical presentation of the patient with MS.

To mitigate the challenges of comorbidity, it is important to monitor the occurrence of comorbidities in the MS population and to describe the medical and the social consequences of these different comorbidities.

The review focuses on providing current evidence regarding the presence of comorbidities in the MS population as reported from different countries and the influence of comorbidities on clinical features, course of the disease, and therapeutic challenges in MS.

### Searching Strategy and Selection Criteria

We identified relevant articles in English for this review by searching PubMed, with no language restrictions, for articles published between January 1, 2014 and May 31, 2020 and reference lists from relevant articles. We used the search terms “multiple sclerosis” (all fields) AND “comorbidity” (all fields) AND [“epidemiology” (all fields) OR “real-world data” (all fields)]. Preferentially, we chose references published within the past 6 years but also included older key or landmark studies in the field. The final reference list was made based on relevance to the theme of this review.

## Investigating Comorbidities in Populations With Ms

To provide valid answers to questions about comorbidities and their impact in a certain disease, it is crucial to find the appropriate data source addressing specific scientific questions (6). To assess a reliable data source, a comprehensive knowledge about the purpose and the method of data collection is needed. The prevalence and the incidence of different comorbidities in a population with MS are addressed using disease registries, administrative databases, or patient surveys, which all have their strengths and weaknesses.

The quality of a disease registry is based on the quality of data fed into it and all the dynamic processes involved in updating it. Administrative databases can provide reliable results, but the performance of comorbidity measures depends on whether all secondary diagnoses are reported and on whether the validity of the diagnoses addressed are verified beforehand (7, 8). Patient surveys are an important source of primary data collection, although the issue of selection bias has to be addressed (9).

An international study assessing self-reported health outcomes and lifestyle behaviors, which recruited participants with MS through social media, MS societies, and websites to answer online questionnaires, found that obesity and most other comorbid disorders were significantly more prevalent in North America than in Australia, New Zealand, and Europe. Obese participants were more likely to have other comorbidities, especially diabetes [odds ratio (OR) 4.8], high blood pressure (OR 4.5), and depression (OR 2.2) (10).

### Comorbidities in MS

Certain comorbidities are more prevalent in people with MS. According to a comprehensive systematic review of 249 articles, the most prevalent comorbidities in MS are depression (23.7%), anxiety (21.9%), hypertension (18.6%), hypercholesterolemia (10.9%), and chronic lung disease (10%) (3).

After the publication of recommendations for observational studies in 2016 (11), which was the outcome of an international workshop, the number of studies investigating the subject of comorbidities increased, particularly for some disease categories.

#### Vascular Comorbidities

Compared with the general population, vascular comorbidities are shown to be more prevalent in the population with MS, including hypertension, hyperlipidemia (12), and ischemic heart disease (13).

A nationwide population-based study including all Danes with clinical onset of MS between 1980 and 2005, linking data from The Danish Multiple Sclerosis Registry to other population-based disease registries, showed a higher occurrence of cerebro- and cardiovascular comorbidities compared with a population without MS matched for all available relevant variables to avoid confounding effects (14).

Results from a population-based study on administrative data from Canada showed that the risk of ischemic heart disease was higher among persons with MS, especially in men aged 20–44 years, compared to the matched population. This increased risk was attenuated later in life (15).

A higher risk of cardiovascular diseases among persons with MS was also confirmed by a registry study from Sweden. Moreover, persons with MS had an overall elevated relative risk for deep vein thrombosis; those with primary progressive MS had a 3-fold higher risk, followed by those with secondary progressive MS and relapsing–remitting multiple sclerosis (RRMS) (16).

A large population-based matched cohort study compared over 12,200 persons with MS registered in the Clinical Practice Research Datalink in England with close to 73,000 controls. Over an 11-years period, patients with MS had 28% increased risk of acute coronary syndrome, 59% increased risk of cerebrovascular disease, and 32% increased risk of any macrovascular disease, which was not completely accounted for by traditional vascular risk factors (17). According to a recent systematic review and meta-analysis, stroke, and ischemic cerebrovascular event occur more frequently in patients with MS (18), although there is a gap of knowledge regarding the extent of the risk and the etiological association with MS.

#### Autoimmune Comorbidities

Autoimmune diseases are relatively rare, but their coexistence with MS is reported by several articles, although a small sample size is a common issue in these studies.

In particular, the coexistence of type 1 diabetes mellitus (DM) is supported by several publications (19). A Danish population-based cohort study found a 3-fold-high incidence rate for MS in the population with DM type 1 (20), which is in line with a case–control approach also based on Danish registries (21).

Studies on DM and MS show a significant heterogeneity, and many studies do not distinguish between DM types 1 and 2, although there are major etiological differences. While the risk of developing type 2 DM mostly depends on lifestyle factors, type 1 DM is considered as an autoimmune disease and thus may share some etiological components with MS. A large database study including ~98% of the pediatric diabetic population in Germany and Austria below the age of 21 years showed a considerably higher risk of co-occurrence of MS in a pediatric and adolescent diabetic population (22).

Thyroid disorders also seem to affect persons with MS more than the general population (21, 23). However, thyroid dysfunction is a known side effect of some DMTs, particularly alemtuzumab (24) and, to a lesser extent, interferon-β (25). Therefore, the part of the autoimmune burden in MS that is independent of DMT-induced thyroid diseases is difficult to establish, but it is important to assess the true causal association.

Other autoimmune diseases, such as inflammatory bowel disease (23) and psoriasis (23), were also found to be more prevalent in the population with MS. A recent systematic review found that the risk of inflammatory bowel disease is 50% in the MS population, and the risk of MS has the same magnitude in persons with inflammatory bowel disease with no apparent differences between ulcerative colitis and Crohn's disease (26).

The issue of asthmatic bronchitis in persons with MS is characterized by some inconsistencies. Previous studies have reported an inverse association between asthma and MS (27, 28), which was not supported by other studies (29). A recently published population-based, cross-sectional study of electronic health record information on 56.6 million Americans showed that the prevalence of asthmatic bronchitis was three times higher among those with MS than in the general population, particularly in the young and the elderly, which differed from the fairly uniform distribution in the general population (30). The reported prevalence of 67 cases per 1,000 persons with MS is considerably higher than previous estimates (29, 31). It is uncertain whether this difference is due to the underlying study population, the study design, the diagnostic criteria, and the differentiation between asthma and obstructive pulmonary disease, and therefore potential limitations should be considered when interpreting these findings. The validity of the diagnostic codes recorded in administrative databases is key consideration in the reliability of such studies. In the recent study based on the Explorys database, which extracts clinical, financial, and operational data, case definition was based on the combination of a diagnosis of MS and a diagnosis of asthma at any time without reporting the validity of the diagnostic data used. As the diagnostic procedure for the two conditions is different, the results may be subject for bias, and the risk of misdiagnosis of MS and an overestimation of asthma cannot be excluded (30).

The coexistence of autoimmune disorders with MS is important, regarding pathophysiological reliable estimates on prevalence and incidence.

#### Cancer

In a comprehensive review, Marrie et al. found that the risk of meningioma and urinary system cancers was greater than expected, while the incidence of pancreatic, ovarian, prostate, and testicular cancers was lower than expected compared to the general population (32).

The association between MS and cancer has long been investigated and led to conflicting results (33, 34). A decreased risk for some types of cancer in the population with MS with clinical onset between 1980 and 2005 was reported by linking multiple, independent Danish nationwide registries (28). A recent Danish study based on linkage of medical registers confirmed that the incidence of cancer in MS patients was similar to the background population, and persons with MS did not have increased cancer-specific mortality (35). This is generally consistent with prior results that did not suggest any different risk of malignancy in MS compared to the general population (33, 36, 37). There are also publications reporting a higher overall incidence of cancer in patients with MS compared to control populations. A large, long-term analysis based on patient records from 6,883 persons with MS born between 1930 and 1979, who were registered with various Norwegian MS and Cancer Registries, suggests that persons with MS may have a greater overall risk of 14% of developing cancer than the general population, with a higher risk of 66% for cancer in respiratory organs, 51% in urinary organs, and risk of 52% for cancer of the central nervous system compared with the non-MS population (38). A nationwide register-based Swedish study found that the cancer rates were overall similar to the rates among the general population, with no evidence of an increased risk for those treated with rituximab or natalizumab but with a borderline significant increased risk of invasive cancer for patients treated with fingolimod, compared to both the general population and the MS population treated with rituximab (39). A previous Swedish study showed increased mortality risk following a cancer diagnosis among persons with MS compared to those without cancer, but this risk was lower than in the general population with cancer (40).

It is important to estimate the incidence of malignancies in MS patients followed up for a long period and to differentiate between untreated patients and patients treated with immunomodulatory or immunosuppressant drugs as it cannot be excluded that the new highly efficacious DMTs increase the risk of malignancies.

In conclusion, the risk of cancer in the MS population needs further clarification; it cannot be excluded that patients with MS have a higher risk for certain malignancies, and the risk may be associated with some disease-modifying treatments.

#### Psychiatric Comorbidities

The prevalence of psychiatric comorbidities in the systematic review by Marrie et al. (41) was rather high (i.e., 23.7% for depression, 21.9% for anxiety, and 5.83% for bipolar disorder).

Depression and anxiety are thought by some to be symptoms of multiple sclerosis, but it is difficult to disentangle the direct effects of the disease upon mood (42).

Estimates of the specific incidence and the prevalence of depression have varied widely in the MS literature due to the heterogeneity of data sources and the definition of depression (42). The comparability of the studies is limited by the assessment methods which range from disease registries to administrative case definitions and different questionnaires that could account for differences in findings between studies. Depression was only present in 5–10% of patients with MS in a retrospective database analysis using data from a large random sample from the proprietary IMS Health Real World Data Adjudicated Claims-US database (43). Using population-based health claims data from Manitoba, Canada, the lifetime and the annual prevalence of depression and anxiety disorders were higher in the MS population than in a matched general population. The annual prevalence ratio of depression was 1.77 (95% CI, 1.64–1.91) and of anxiety disorders was 1.46 (95% CI, 1.35–1.58) (44). A nested-case cohort study based on Danish disease registries reported an OR of 1.4 (95% CI, 1.18–1.62) for depression in the MS population compared with controls and for anxiety an OR of 1.1 (95% CI, 0.78–1.43) (45). The higher risk for developing depression when having MS was supported by a Swedish registry study [hazard ratio (HR) 1.9; 95% CI, 1.7–2.0] (46). Depressive symptoms are influenced by a number of factors such as body mass index, number of comorbidities, fatigue, and disability (significantly predictive of positive depression screen), while being married, employed, and educated were associated with a lower risk of depression among people with MS (47).

Depression and anxiety were prevalent in 27 and 40%, respectively, in the Australian Multiple Sclerosis Longitudinal Study using the Hospital Anxiety and Depression Scale (HADS), and 20% of the participants had both disorders (48). Healthier lifestyle factors were associated with a lower prevalence of depression and also milder severity of depression but not anxiety (48). Nearly 30% of the 2,544 participants had anxiety as assessed with the HADS in a cross-sectional study conducted in the MS outpatient clinic of the University of Calgary, underlining the substantial burden of anxiety for those with MS (49).

A population-based Swedish study investigating the risk of MS in persons with psychiatric diagnoses found a considerably higher risk of MS in patients with bipolar disorder, with a hazard ratio (HR) of 1.8 (95% CI, 1.6–2.2), and a lower risk for schizophrenia (HR 0.6, 95% CI, 0.4–0.9) (46).

The relationship between psychiatric comorbidities and MS is likely to be complex. An Italian study of 405 RRMS patients found higher rates of depression and anxiety on the Beck Depression Inventory II and State/Trait Anxiety Inventory among patients with MS with radiological activity on brain magnetic imaging (MRI). In a subset of 111 treatment-naive patients, a positive correlation between depressive symptoms and the level of pro-inflammatory cytokines was detected in the cerebrospinal fluid (50).

There are few reports on the treatment of psychiatric comorbidities. In a recent Canadian study, 85.7% of persons with MS and diagnosed with depression were treated, of whom 65% were taking antidepressant medications (51), which is higher than in previous reports (52).

In conclusion, neuropsychiatric symptoms such as depression, dysphoria, and cognitive impairment are a part of MS symptomatology and can also occur as comorbidity. Both MS and depression share several symptoms, such as fatigue, poor concentration, and sleep problems, which can further complicate the diagnosis of depression in MS.

#### Other Comorbidities

Back pain is not commonly studied, but it was the most commonly reported comorbidity by 36.2% of the participants in the “Health Outcomes and Lifestyle in a Sample of People With Multiple Sclerosis” study based on self-reported questionnaires (10).

Migraine is twice as likely to occur in persons with MS than in those without (53) and is suspected to increase the relapse rate (54).

Chronic lung diseases are common in the MS population and, according to a large Canadian study, it is considerably higher among persons younger than 45 years of age, but the authors did not distinguish between asthma and chronic obstructive lung disease (55).

Sleep disorders have been commonly reported in people with MS, with a prevalence rate higher than expected (56), particularly, sleep-related breathing disorder, insomnia, and restless legs syndrome (57). However, the association between poor sleep, fatigue, depression, and anxiety is bidirectional (58, 59). While insomnia may be secondary to other disabling symptoms such as nocturia or pain or comorbid anxiety and depression, injury of specific areas of the central nervous system can be associated with sleep-related breathing disorder (60), restless leg syndrome (61), or rarely narcolepsy (62). Sleep disturbances affect cognitive functions and can thereby exacerbate the existing cognitive dysfunction in persons with MS (63).

Little is known about the coexistence of MS and Alzheimer's disease (AD) as there is no evidence of a higher risk in persons with MS than in the background population (64), but due to the increased longevity, people with MS will be affected by the same age-related diseases as the general aging population. A recent study compared cognition in older patients with MS to patients with AD and with patients diagnosed with mild cognitive impairment and found that MS is not associated with impairment of memory consolidation compared with AD, while there may be some overlap between MS and the prodromal stages of AD (65). MS is considered as a white matter disease, but growing evidence indicates that in MS the gray matter is also affected and that pathology correlates with cognitive function deterioration in MS (66). Therefore, the identification of reliable diagnostic and prognostic biomarkers of cognitive impairment could contribute to a better understanding of the pathogenesis of cognitive deterioration in patients with MS.

### Effects of Comorbidities on Time to Diagnosis, Treatment-Related Topics, Clinical Course, Quality of Life, and Socioeconomic Outcomes

#### Effects on Time of Diagnosis

A nationwide population-based Danish study reported longer diagnostic delays for MS in persons who suffered from cerebrovascular, cardiovascular, and pulmonary disease as well as DM and malignancies (67), supporting the finding reported from the NARCOMS registry on diagnostic delay caused by comorbidities (68).

#### Effects on Treatment-Related Topics

A large Canadian study of 10,698 patients found that the likelihood of starting DMT decreased with an increasing number of comorbid conditions (69). Specific comorbidities such as ischemic heart disease and anxiety were associated with a lower likelihood of DMT initiation (69). Interestingly, comorbidities were not significantly associated with the choice of the first DMT but were significantly associated with a higher risk of switching from the first DMT due to intolerance (70).

The presence of some comorbidities may be a contraindication when prescribing a DMT, (e.g., cardiovascular diseases and malignancies). A pre-existing comorbidity may also influence DMT adherence, persistence, tolerability, and possibly effectiveness. Once a DMT is initiated, the potential to trigger the development of a new disease may further increase the comorbidity burden in the general MS population. A study based on population-based health administrative data in British Columbia, Canada, reported that treatment with IFN-β was associated with a 1.8-fold increase in the risk of stroke, a 1.6-fold increase in the risk of migraine, and a 1.3-fold increase in depression and hematologic abnormalities (54). Therefore, long-term post-authorization safety studies conducted by registries, including a complete and unselected population, are crucial to identify, characterize, or quantify a safety hazard of medicine for MS.

#### Effects on Clinical Course and Quality of Life

The clinical course of MS can be very diverse, and comorbidities may contribute to this heterogeneity. Several comorbid conditions are associated with disability progression, including DM, hypertension, and chronic obstructive pulmonary disease (71). A prospective multicenter cohort study conducted in four Canadian MS clinics reported an increased relapse rate over the next 2 years if the patients had three or more comorbidities at baseline compared to those with no comorbidity (54).

Moreover, the presence of depression was associated with a significantly higher subsequent disability over an average of 10 years of follow-up in women who constituted two-thirds of the investigated population (72). The impact of depression was also investigated in more than 15,000 patients from The Swedish MS registry, which found that MS patients with depression or bipolar disorder showed a significantly higher risk of progression on the Expanded Disability Status Scale (EDSS) score (73).

A cohort study using the NARCOMS Registry found that MS patients with vascular comorbidities at any time during their disease course progressed to an EDSS score of 6 on average 6 years faster than MS patients without a vascular comorbidity (74) and that comorbidities affected the visual disability (75).

Rheumatoid arthritis was associated with more than 3-fold, and anemia had a 2-fold increased hazard of relapse (76).

Interestingly, a study including brain MRI as outcome found that the presence of DM type 1 was associated with a significant reduction in gray matter, particularly cortical gray matter volumes, independent from the demographic and the clinical features of MS (77). Another study found that the presence of autoimmune comorbidities, especially psoriasis, thyroid disease, and DM type 2 comorbidities, was associated with more severe MRI outcomes of neurodegeneration and demyelination (78).

In a study based on self-reported information, a higher number of comorbidities was related to worse quality of life and increased odds of disability (10). Moreover, depression and anxiety can negatively impact on quality of life and other MS symptoms, such as fatigue and cognitive function (79–81).

A Canadian study found that MS patients with one or more comorbidities had a 2-fold higher all-cause hospitalization rate than MS patients without any comorbidity (82). Another Canadian study using administrative data linkage reported that fatigue and the presence of three or more physical comorbidities were significantly associated with higher rates of physician encounters, prescriptions filled, and hospitalizations (83).

#### Effects on Socioeconomic Outcomes

The social and the economic consequences of comorbidity in MS were investigated in a Danish study in which multiple independent, nationwide population-based registries were linked. Interestingly, the presence of any somatic, but not psychiatric, comorbidity increased the risk of broken relationships and increased the odds of low incomes in persons with MS (4). The risk of requiring disability pension was increased in MS persons with comorbidities in a Swedish study using a similar approach (84).

### Effect of Comorbidities on Mortality

The presence of comorbidities is associated with increased mortality. Thormann et al. reported that mortality is increased in persons with MS with coexisting psychiatric, cerebrovascular, cardiovascular, lung, DM, cancer, or Parkinson's disease comorbidity (67). These results confirm the survival disadvantage reported for cerebrovascular diseases among MS patients in a Danish study (85). Additionally, a recently published large study from Finland showed that the mean survival time is lower for MS patients with any circulatory disease (86). Among the NARCOMS registry participants, vascular, mental, and visual comorbidities increased the risk of mortality (87).

## Discussion

Comorbidities are common in the MS population and may account for some of the heterogeneity, including diagnostic delay, clinical presentation, disability progression, and the rate of health care utilization.

The relationship between comorbidities and MS is complex and it may due to (1) direct causal relationship, common risk factors, or (2) a secondary association (e.g., increased risk of cardiovascular diseases or infections in immobile MS patients or less exposure to certain factors in MS patients associated with the risk of developing another specific disease).

Investigating comorbidities may lead to a better understanding of the shared etiological pathways of coexisting diseases. Understanding the pathophysiological mechanisms underlying inverse comorbidity could provide insights into factors protecting against MS (28).

The temporal relationship between the onset of comorbidities in relation to the onset and the diagnosis of MS is difficult to determine due to uncertainty of the onset of the MS disease in relation to the onset of clinical symptoms and several possible confounding factors and bias, particularly ascertainment bias. A large study using the UK Clinical Practice Research Datalink reported that the Charlson comorbidity burden (88) was already higher at the time of MS diagnosis and the risk of new comorbid conditions after the diagnosis of incident MS did not differ from that in controls (89). Comorbidities were shown to delay the diagnosis of MS (67, 68), which further complicates the temporal association of the coexisting diseases and may contribute to the increased disability at the diagnosis of MS.

It is useful to separate the presence of symptoms being a part of MS itself from comorbidities that are more prevalent in the MS population, and this is particularly challenging in case of depression and anxiety. Psychiatric comorbidities may develop even before the diagnosis of MS (45), and, hence, early diagnosis of psychiatric conditions is important in order to prevent suicide, which is higher in the 1st years after the diagnosis of MS (90). Depression and anxiety may be associated with MS-related damage of the central nervous system (CNS), response to chronic illness, or possibly side effects of certain disease-modifying treatments. Furthermore, the complex relationship between anxiety and depression and their mediating role of fatigue and cognitive symptoms are difficult to detangle (91). Anxiety and depression are associated with reduced cognitive function in MS (89), so the management of symptoms and of the factors associated with a higher risk of developing these conditions is important to mitigate their effect on cognition.

Comorbidities have important clinical implications, especially when coexisting diseases affect the CNS due to the overlapping symptoms. Furthermore, coexisting type 1 diabetes is reported to be associated with an increase in brain atrophy in persons with MS (92).

The prevalence of comorbidities increases with age, and more elderly patients with MS may suffer of age-related comorbidities (93). The burden of comorbidities, particularly of chronic somatic diseases, is significantly more likely in the older age groups (43), which represents a challenge in the aging population with MS as comorbidities apparently modify disease activity, worsen disability and chronic symptoms, and, overall, negatively affect the quality of life.

The issues related to comorbidities should be a part of patient counseling, especially regarding modifiable lifestyle factors, because preventing comorbidities can alleviate the burden of MS. Interestingly, the risk of macrovascular diseases, including acute coronary syndrome and cerebrovascular disease, was higher in people with MS even after controlling for vascular risk factors and socioeconomic characteristics (17).

However, overweight, smoking, and physical inactivity are strongly associated with diabetes and vascular diseases. Therefore, persons with MS should be encouraged for positive health behavior (94). Ever-smoking was associated with a higher burden of MS typical brain lesions on MRI compared with matched controls without vascular risk factors (95). A 12-weeks course of high-intensity aerobic exercise, in combination with resistance training, was found to improve glucose tolerance in persons with MS (96). According to a systematic review, physical training interventions in persons with MS improve the vascular risk factors and may be considered as a therapeutic strategy for managing vascular comorbidities (97). There is strong evidence that smoking—apart from being a strong risk factor for vascular and pulmonary diseases—increases the EDSS score (98), so smoking cessation is strongly recommended.

The role of comorbidities in rehabilitation remains a poorly investigated issue and represents an important gap of knowledge. A comprehensive review of comorbidities on the effects of neurorehabilitation interventions in multiple sclerosis found that, of the selected 54 RCTs, 40 excluded individuals with comorbidities, and only two reported on the comorbidity of the enrolled participants. Strikingly, none of the 14 RCTs that allowed MS patients with comorbidity to be included examined comorbidity as a moderator or mediator of intervention outcomes, thereby compromising the effectiveness of the rehabilitation intervention (99).

The impact of comorbidities on clinical outcomes may contribute to the discrepancy in treatment outcomes and reported adverse events in randomized controlled trials with a highly selected patient population and observational studies in a non-selected but highly representative population (100).

With the approval of new DMTs and thereby an increased number of consecutive treatments in the same patient, the potential of developing new comorbid conditions is increased (101). Therefore, in order to build evidence about the long-term safety of DMTs, long-term monitoring of adverse events is crucial for distinguishing any association with a DMT or a specific sequencing of therapies. Following an unselected population with MS in a real-world setting in order to capture all coexisting conditions is important for distinguishing between spontaneous comorbidities and similar symptoms caused by rare unrecognized adverse events.

In conclusion, comorbidities increase the complexity of patient management and have health, social, and economic consequences for people with MS. Therefore, more effort is needed to understand the prevalence and the incidence of comorbidities that must be investigated in different age groups as the implications for treatment, work productivity, and quality of life can vary. When assessing comorbidities, the quality of the data sources applied to generate evidence is crucial for the validity of the results. High-quality disease registries and administrative databases covering an entire population can therefore contribute, in a greater extent, to the elucidation of the scientific questions regarding comorbidities.

## Author Contributions

MM: study concept, design, literature search, and primary author of the manuscript. PS: study concept, design, and critical revision of manuscript. All authors contributed to the article and approved the submitted version.

## Conflict of Interest

The authors declare that the research was conducted in the absence of any commercial or financial relationships that could be construed as a potential conflict of interest.
